# Supplementary material for: Are carbon emissions trading and green financial instruments synergistic? -Comprehensive quantitative research based on content analysis
Source: PLoS One. 2024 Mar 7;19(3):e0298601. doi: 10.1371/journal.pone.0298601 (PMC10919601; doi:10.1371/journal.pone.0298601)
Supplement: S1 Data — (ZIP) [file pone.0298601.s001.zip › Data/地级市Z_F工作报告.zip!/╕ú╜¿/─╧╞╜╩╨/└┤╫╘╣1⁄2╓┌║┼í╛┬φ┐╦╩2╛▌═°í┐_2015.docx]

　各位代表：

　　现在，我代表南平市人民政府向大会报告政府工作，请予审议，并请市政协各位委员和其他列席人员提出意见。

　　一、2014年工作回顾

　　2014年，在省委、省政府和市委的正确领导下，我市各级政府认真学习贯彻党的十八大、十八届三中、四中全会和习近平总书记系列重要讲话特别是来闽考察重要讲话精神，围绕创建全国绿色发展示范区目标，按照“发展要赶超、改革要争先、稳定要创优、环境要做美、民生要做好、作风要过硬”的要求，全力稳增长、调结构、促改革、惠民生，经济运行稳中有进。初步统计，全市生产总值1232.6亿元，增长9.6%；公共财政总收入118.3亿元，增长10.9%，地方公共财政收入81亿元，增长13.1%；全社会固定资产投资1481.3亿元，增长22%；外贸出口14.7亿美元，下降4.3%；验资口径实际利用外资1.2亿美元，增长14.3%；旅游接待总人数2503万人次，旅游总收入312.5亿元，分别增长18.9%、20.9%；城镇登记失业率3.1%；社会消费品零售总额451.7亿元，增长12.9%；居民消费价格总水平上涨2%；人口自然增长率6.6‰；城镇居民人均可支配收入24074元，增长8.8%；农民人均可支配收入11252元，增长11.5%，基本完成市四届人大三次会议确定的年度目标任务。

　　（一）绿色发展效应日益显现。坚定不移推进绿色发展，主动应对经济下行压力增大等复杂形势，及时出台并落实好促进实体经济发展系列政策措施，积极向上争取政策和资金支持，成功获批国家水生态文明城市试点、绿色循环低碳交通运输城市区域性试点。持续突破交通、平台、项目、招商等重点，合福高铁南平段、南三龙铁路、4条高速公路和一批国省道改造工程加快建设，衢宁铁路开工建设，与沿海的时空距离逐步缩短；突出打造城市、产业园区、旅游景区三大平台，经济发展的承载能力增强；绿色发展示范项目、迎接高铁时代到来等重大项目加快建设，投资规模扩大、结构优化；招商环境持续改善，成功举办第十七届“5·13”投洽会、第八届茶博会，引进总投资2000万元以上合同项目1175项、总投资2573亿元，项目开工率75.8%，经济发展质量提升、后劲增强。

　　（二）优势特色产业加速集聚。实施食品加工、旅游养生、机电制造、生物医药等千亿五百亿产业行动计划，完善提升市本级三大产业园区和各县（市、区）专业园区，实施产业龙头促进计划，8家企业被列为省级龙头企业，新增产值超亿元企业70家。实施亿元以上重点技改项目103项，竣工投产项目26个，新认定国家级高新技术企业7家。打造融资、技术、市场服务平台，设立增信资金池，建立封闭转贷机制，有效降低企业融资成本。加快发展电子商务产业，全市电商企业销售额突破60亿元。全力实施迎接高铁时代旅游工作行动方案，新增国家4A级旅游景区2家、3A级5家，建成4个休闲集镇、6个特色村，旅游承载能力持续增强。产业转型升级步伐加快，特色进一步凸显。

　　（三）“三农”基层基础更加扎实。粮食生产保持稳定，茶叶、果蔬、食用菌、烟叶等特色农产品持续增长，培育规模以上农产品加工企业473家，农林牧渔业总产值462.8亿元、增长5.3%。推进土地流转67.9万亩，新增农民专业合作社455个，培育家庭农场582个，其中12个被列入省级示范场。农业“五新”示范推广374项，新增设施农业1.2万亩，新增5个省级林下经济示范县。完成水利建设投资24亿元，新开工重点水利项目5个，水库除险加固45座，解决28.8万农村人口饮水安全问题。

　　实施农村路网建设与改造416公里、农村公路安保工程1215公里，完成危桥改造64座。完成造福工程危房改造6810户，5个省级扶贫开发重点县对接帮扶项目267个，落实资金16.2亿元。新增193个村财年收入10万元以上的建制村。

　　（四）新区新城建设全面提速。经过多年努力，我市部分行政区划调整方案于2014年5月成功获批。我们紧紧把握这个重大机遇，统筹推进武夷新区和延平新城建设。武夷新区各片区控规基本完成、基础设施立体交叉推进，水之廊景观主体工程基本完成，赤岸、南林统建房和四所学校13栋建筑封顶，快速通道高铁武夷山东站至公馆大桥段开工建设，崇阳溪旅游景观工程绿道及马拉松赛道将口示范段基本完成，核心区完成投资104亿元、增长38%，引进大项目30个、总投资285.6亿元。延平旧城改造和新城建设同步推进，城区控规基本完成全覆盖，水东新大桥、朱熹路提升改造工程、高铁站前片区、夏道大桥、成功路和东坑岭隧道开挖工程基本完工，城区体育公园启动建设；产业启动区3000亩平台完工，太阳电缆城一期工程投产，新城完成投资63.9亿元、增长38.1%，引进项目63个、总投资204亿元。

　　（五）共建美丽南平取得成效。新型城镇化建设步伐加快，光泽生态食品城、邵武产城融合试点和4个省级、10个市级小城镇综合改革试点镇扎实推进。实施宜居环境建设行动计划，加快推进城市建设“六个一”工程、“三边三节点”和市政设施“五千工程”建设，启动宜居社区创建工作，深化“点线面”攻坚，拆除“两违”面积351万平方米，城市面貌进一步改观。实施共建美丽南平“百千万”工程，131个美丽乡村建设全面铺开，顺昌张墩村荣获第八届“中国十佳小康村”，南平至武夷山高速公路森林生态和沿线村庄景观整治全面启动，1592个建制村建立卫生保洁长效机制，万人保洁“南平经验”成为全省典型。

　　（六）重点领域改革深入推进。完成本轮市县政府机构改革、食品药品监管体制改革和工商注册资本登记制度改革，稳步推进事业单位分类改革。率先在全省公布市、县两级行政审批事项与公共服务事项目录清单，保留市本级行政审批事项210个，下降15.3%。创新旅游发展机制，运用互联网思维开展“一元门票游大武夷”活动，达到大宣传、大带动、大演练的效果，实现观光旅游向旅游产业转变、小武夷旅游向大武夷旅游转变。市属国有企业加快整合，组建武夷集团等9家国有企业，累计融资170亿元。全市以药品耗材零差率为切入点的公立医院综合改革全面推开，组建武夷总医院，成功托管政和县医院，医疗水平进一步提升。创新土地林权流转抵押机制，成立流转抵押服务平台，推动农业规模化、组织化、集约化发展。积极融入“一带一路”战略，成功召开第三届“万里茶道”与城市发展中蒙俄市长圆桌会议。

　　（七）生态环境质量持续优化。8个县（市）获批省级生态县，新创建国家级生态乡镇39个、省级生态乡镇6个。造林绿化37.1万亩，加强茶山整治，治理水土流失面积32.8万亩。筛选实施第一批18个总投资102亿元的节能减排典型示范项目，完成55个工业减排项目、150个畜禽养殖治理项目、25个闽江流域重点整治项目治理任务，提前一年实现“十二五”节能减排目标。全市主要流域水质功能达标率100%，城市集中式饮用水源水质达标率100%，城市环境空气质量优良天数比例99.7%，南平的水更清、山更绿、空气更清新。

　　（八）社会事业发展步伐加快。21项为民办实事项目基本完成。新增城镇就业2.45万人，农村劳动力转移就业5.52万人。社会保障体系建设稳步推进，城乡居民养老保险制度一体化全面实施，基本医疗保险制度改革持续深化。新建2个县级社会福利中心、8所乡镇敬老院和147个农村幸福院。保障性安居工程开工5298套、基本建成7924套，均超额完成省上下达任务。4个县（市）通过全国义务教育发展基本均衡县认定，中小学校扩容改造新增学位3200个，新增公办幼儿园10所、义务教育标准化学校21所，武夷学院等高校办学水平进一步提升。精神文明创建活动持续深化，县级公共图书馆、文化馆、博物馆和乡镇综合文化站建设取得成效。成功举办第七届科技成果交易会，科技成果转化能力提升，知识产权工作不断强化。人才强市战略深入实施，人才引进力度加大。卫生“健康云”服务平台建设成效明显，登革热等重大疾病有效防控。群众体育和竞技体育运动蓬勃开展，举办武夷山国际马拉松等赛事，建成全国青运会（南平赛区）综合馆。人口计生综合治理“三高”取得成效，计划生育工作整体水平提升。妇女儿童、老龄和残疾人工作得到加强，社会福利和慈善事业稳步发展。“平安南平”建设持续深化，医患纠纷“南平解法”长效机制不断完善，“詹红荔工作法”、和谐征迁法全面推行，信访积案有效化解，社会保持安定稳定。建立安全生产“党政同责、一岗双责”机制，安全生产专项整治、道路交通安全综合整治深入开展，安全生产形势稳定好转。民族宗教、外事侨务、气象防震、档案方志、老区库区等工作继续加强。推进军民融合深度发展，军政军民关系融洽和谐。

　　（九）政府作风建设得到加强。深入开展党的群众路线教育实践活动，严格落实中央八项规定精神，加强“四风”突出问题专项整治，全面停止新建楼堂馆所，清理超标办公用房，狠刹公款“吃喝风”，整治公车私用、庸懒散奢、“吃拿卡要”现象，全市“三公”经费支出下降23.5%，考核检查、评比表彰和认定类项目减少73%。持续深化“三级联动、重心下移”工作机制，全面推广“民生110”网格化服务，打通联系服务群众“最后一公里”。自觉接受人大和政协监督，办理人大代表议案1件、建议160件，满意率为100%，办理政协委员提案265件，满意率为99.2%。虚心听取民主党派、工商联、无党派人士和社会各界的意见建议，广泛接受群众监督。实施机关效能建设工作条例，“马上就办”持续深化，绩效管理有效拓展。行政监察和审计监督力度加大，信息公开和政务公开制度不断完善，廉政建设和反腐败工作扎实推进。

　　成绩来之不易，这是省委、省政府正确领导、亲切关怀的结果，是市委总揽全局、科学决策的结果，是人大和政协大力支持、有效监督的结果，是全市人民凝心聚力、奋力拼搏的结果。在此，我代表南平市人民政府，向全市人民，向全体人大代表、政协委员，向各民主党派、工商联、各人民团体、无党派人士、离退休老同志和社会各界人士，向中央、省直驻延机构和驻军部队、武警官兵、公安民警，向对口帮扶部门和地区，向关心支持南平发展的港澳台同胞、海外侨胞、国际友人，表示衷心的感谢！

　　在充分肯定成绩的同时，我们也清醒地认识到发展还存在不少困难和问题，主要是：经济总量较小，产业竞争力和自主创新能力偏弱；龙头企业带动力不强，部分企业经营困难，融资难、融资贵现象比较突出；城乡建设管理水平还有待提高；公共服务设施欠账较多；政府职能转变还不到位，法治政府建设有待加强。我们必须高度重视这些问题，采取有效措施认真加以解决。

　　二、扎实做好2015年工作

　　今年是落实科学发展跨越发展行动计划的第一年，也是实施“十二五”规划的最后一年。我们必须以新状态适应新常态，继续保持艰苦奋斗的良好作风和奋发有为的精神状态，坚定信心、抢抓机遇，自加压力、主动作为，加快推动科学发展跨越发展。

　　政府工作的总体要求是：全面落实党的十八大和十八届三中、四中全会精神，以邓小平理论、“三个代表”重要思想、科学发展观为指导，深入学习贯彻习近平总书记系列重要讲话和来闽考察重要讲话精神，认真落实中央支持福建进一步加快经济社会发展的一系列政策措施，按照市委四届七次、八次全会部署，坚持稳中求进、好中求快，坚持绿色发展，主动适应经济发展新常态，全力做好迎接高铁到来各项工作，深化改革开放、突出创新驱动、做强产业支撑、加强民生保障，努力建设机制活、产业优、百姓富、生态美的新南平。

　　全市经济社会发展的主要预期目标是：地区生产总值增长11%，公共财政总收入和地方公共财政收入均增长11%，全社会固定资产投资增长22%，外贸出口增长8%，验资口径实际利用外资增长10%，旅游接待人数和收入均增长30%，城镇登记失业率控制在4.4%以内，社会消费品零售总额增长15%，居民消费价格总水平涨幅控制在3.5%左右，人口自然增长率控制在9.5‰以内，城镇居民人均可支配收入增长10%，农民人均可支配收入增长11.5%，完成单位生产总值能耗、化学需氧量、二氧化硫、氨氮、氮氧化物等年度节能减排任务。

　　重点抓好以下九个方面的工作：

　　（一）发挥投资拉动作用，落实科学发展跨越发展行动计划

　　加快实施重点项目。认真落实行动计划确定的722项总投资7166亿元的重大项目，全年实施重点项目350个，总投资2645亿元，其中在建项目269个，年度计划投资408亿元。用好用足中央、省上扶持南平发展的政策，继续策划生成一批带动力强的重大产业项目、基础设施项目和民生工程项目，形成策划一批、储备一批、开工一批、投产一批的项目滚动发展格局。

　　持续完善基础设施。建成京福高铁南平段，加快建设南三龙铁路、衢宁铁路，扎实推进浦建龙梅铁路前期工作。加大武夷山机场航线航班培育力度，积极推进武夷山新机场前期工作。建成京台高速公路南平段和邵光、延顺高速公路，新增通车里程175公里，实现“县县通高速”；加快建设南平联络线和顺邵高速公路，加快推进武夷新区绕城高速、厦沙高速南平连接线前期工作。加快国省道干线和农村公路改造升级，建成武夷新区、南平安济等5个客货运及公交场站。加快中海油海西天然气管网二期工程南平段、华电邵武火电厂扩建等项目建设。加快4G移动基础网络和光网城市建设。

　　强化项目要素保障。加大协调服务力度，开通审批绿色通道，推行项目交叉并联审批，深化行政审批“三集中”改革。健全政银企对接机制，争取银行业金融机构更多的支持，扩大信贷规模。推广PPP等建设运营模式，创新方法筹措建设资金。坚持和谐征迁，积极争取用林用地指标，保障重点项目用地需要。

　　（二）积极迎接高铁到来，推动第三产业快速发展

　　做好迎高铁到来各项准备。全力实施迎接高铁时代到来行动方案，加快推进与高铁配套衔接的交通、旅游、城建、物流等重点项目建设，确保与高铁同步建成投入使用。抢抓高铁通车机遇，把发展旅游业和现代服务业作为主攻方向，围绕“进得来、行得畅、容得下、留得住、玩得好、能消费”的工作要求，完善吃住行游购娱等要素，加快现有服务业企业提档升级，培育一批规模以上服务业企业，推动第三产业实现有速度、有质量的发展。

　　构建大武夷旅游格局。进一步整合全市旅游资源，创新宣传营销模式，持续开展“一元门票游大武夷”活动，加强市内旅游联盟和跨区域跨行业合作，完善旅游运作机制，打响大武夷旅游品牌。精心打造以武夷山为龙头，有机串联各县（市、区）特色景区景点的旅游交通网络体系和旅游线路，统一策划营销运营。加快推进武夷山五夫、下梅、邵武和平古镇、建阳考亭、延平溪源庵、政和佛子山等旅游项目建设，建成4条旅游景区公路，启动3个国家5A级旅游景区创建工作，力争全年新增8个以上国家4A级旅游景区、20个以上旅游休闲集镇和特色村。加快智慧旅游建设，加强旅游市场监管，推进景区、旅行社、旅游住宿等要素标准化、规范化、智能化建设，促进旅游产业转型升级。

　　加快发展现代服务业。推进市本级三大产业组团综合性物流园区建设，各县（市、区）规划建设一个物流产业园，加快城市商贸综合体和专业街区建设。支持群众刚性住房需求，推动房地产业持续健康发展。加快发展电子商务产业，推进武夷山紫阳古城、延平新城电子商务产业园建设，鼓励个人电商创业，扶持一批电商企业发展壮大。培育发展新兴服务业，吸引企业集团总部、交易平台、金融保险等机构进驻南平，加快发展会展经济、医疗保健、养老养生、体育健身以及信息、咨询、中介及其他服务业。

　　（三）坚持大招商招大商，培育壮大优势主导产业

　　提升招商引资实效。整合招商资源，推动综合招商与小分队招商、产业链招商与专业园区招商相结合，实施“回归工程”，加强与省属国企对接，积极开展对台招商。主动参与“6·18”、“9·8”等重大展会，重点办好“5·13”投洽会和“11·16”茶博会，力争全年招商合同项目总投资达3000亿元。落实招商优惠政策，全力抓好项目落地，对引进重大项目实行“一对一”跟踪服务，确保当年引进项目开工率70%以上。

　　做大做优特色产业。推进食品加工、旅游养生、机电制造、竹加工、生物医药等千亿五百亿产业行动计划，围绕“5+3”产业，进一步明确各县（市、区）的主攻方向，不断延伸产业链条。加快市本级三大产业组团和各县（市、区）专业园区建设，完善污水处理等基础设施和公共服务设施，促进产业集聚发展。按照世界眼光、国际标准、闽北特色的要求，建立产品标准化体系、质量安全可追溯体系和商标品牌保护体系，打造武夷食品、武夷旅游、武夷茗茶、武夷竹业等品牌，提升产业竞争力。

　　实施龙头促进计划。落实工业稳增长、扶持现有企业增资扩产、帮扶企业解困等政策，鼓励企业通过上市融资、发行债券和战略重组等方式快速扩张，支持中小企业在新三板上市。扶持圣农集团、太阳电缆、南孚、欧浦登光学、福矛酒业等一批现有企业做大做强，加大对小微企业的扶持力度，实施80个总投资221亿元的重点技改项目，新增产值亿元以上企业65家、5亿元以上企业5家。突出品牌带动，新注册商标500件以上，新申报福建名牌产品15项，新增中国驰名商标2件、省著名商标30个。

　　（四）建设特色现代农业，着力转变农业发展方式

　　提升农业规模化产业化水平。抓好粮食产能区和高产示范片建设，强化粮食安全保障。实施现代农业发展行动计划，加强农业“五新”技术推广，实施种业创新工程，扶持“育繁推一体化”种子示范企业发展。引入工业化、信息化手段，加快建设设施农业基地，推动畜禽养殖业适度规模经营，培育百合花、丹桂、薏米、桔柚、锥栗等地理标志产品和特色农产品，发展笋竹、茶叶、烟叶、油茶、食用菌、中药材等产业和果庄、茶庄、渔庄、山庄等休闲农业，实现三次产业联动发展。

　　改善农村生产生活条件。建设高标准示范农田7万亩。开工建设浦城王家洲、松溪虎洋、政和昌岐洋水库，加快光泽茶富水库前期工作，完成22座病险水库除险加固、20条中小河流综合治理项目。完成农村路网建设改造350公里、危桥改造33座。实施农村饮水安全工程，解决8万农村人口饮水安全问题。

　　创新农业经营方式。放活土地经营权，健全县乡土地流转服务平台，加快推动农村土地承包经营权确权登记颁证工作，完善土地林权流转抵押机制，探索建立农村产权综合交易中心。培育新型职业农民，发展家庭农场100家，规范提升农民合作社，促进农业增效、农民增收。盘活乡村集体资产资源，培育壮大乡村集体经济，新增一批村财年收入10万元以上的建制村。

　　推进科学扶贫精准扶贫。落实扶贫开发政策和对口结对帮扶机制，推进山海协作共建产业园区。坚持“输血”、“造血”并重，支持原中央苏区、革命老区、少数民族地区、偏远山区和库区发展。整合部门扶贫开发资金，抓好产业扶贫、扶贫到户、整村推进、异地搬迁扶贫、基础设施攻坚、公共服务提升等“六大工程”，增强贫困地区自我发展能力，力争全年减少贫困人口3万人。

　　（五）加快建设武夷新区，打造国际旅游养生度假城市

　　优化城市组团布局。围绕建设“世界遗产地、绿色生态城”目标，完成城市设计导则编制，统筹规划“一城六片”，加快推进童游、将口、兴田三大组团建设，科学布局南林核心区，建设低楼层、低密度、厚重感强的建筑，做到依山就势、错落有致，争创全国绿色建筑示范城区和国家园林城市。按照建阳、武夷山与武夷新区一体化发展的要求，统筹谋划城乡规划管理、重大基础设施建设、城市建筑风格风貌和产业发展空间布局，促进教育卫生、文化体育、金融保险、就业保障等公共服务资源共建共享。

　　加快公建设施建设。全线开工快速通道和轨道交通、崇阳溪旅游景观慢道工程，完善核心区城市骨干路网。建成新区四所学校、赤岸统建房、山体公园一期等项目，加快市民广场、新岭统建房建设，开工建设森林生态博物院、云谷小区、文化中心、博物馆、森林公园、医院等项目，完善公共服务配套设施，有序引导人口、城市功能配套及发展要素向核心区集中集聚。

　　推动产业集聚发展。加快发展食品加工、旅游养生、生物医药等产业和新材料、新能源等绿色产业，大力发展文化创意、现代物流、会议会展、体育赛事等现代服务业。加快武夷高新技术产业园区建设，整合市本级三大产业园区、武夷山市园区建设的资源，积极申报创建省级和国家级高新技术产业园区。

　　（六）持续共建美丽南平，构建宜居宜业城乡环境

　　加快延平新城建设。围绕打造“山水宜居之城、活力创业之城”目标，加快新城城市功能区和产业启动区建设，完成高铁站前片区综合配套、南平新大桥北幅桥、滨江北路，开工建设朱熹路五期、从彦路、疏港路、莲花山道路、西芹大桥等路桥，加快城区4个体育公园和延平书院等社会事业设施建设，实现新老城区联动发展。加快电缆城和白炭黑林产化工循环经济园建设，持续推进“退城进园”，推动产城融合发展。

　　做精做美县域城市。围绕“人进城、建好城、管好城”要求，加快邵武、光泽省级新型城镇化试点建设，推进省级、市级小城镇综合改革试点镇和仙阳省级“小城市”培育试点镇建设，就近就地转移农业人口。开展城乡总体规划修编，推进“多规合一”，优化空间布局，同步推进旧城改造和新城建设。持续实施“六个一”工程，各县（市、区）建设或改造提升一条民俗文化街、一个主题文化公园、一个城市商贸综合体、一个4A级旅游景区、一个完整社区、一个专业产业园。继续实施宜居环境建设行动计划、“点线面”攻坚计划和“五千工程”，提升城区“三边三节点”规划和景观整治水平，加强城市管理，推动社区共建共享。

　　丰富美丽乡村内涵。深入实施“百千万”工程，加快打造百个美丽乡村试点，推进高速、高铁、国道两侧千里生态绿廊建设，持续抓好万人保洁工作和“两违”综合治理工作，进一步优化农村人居环境。加大对历史文化资源和古村落、古建筑分级保护力度，对文化底蕴深厚、田园风光秀丽等特色鲜明的村庄，按照A级景区标准同步规划、同步建设，提升美丽乡村建设品位。

　　（七）深入推进改革开放，激发经济社会发展活力

　　深化重点领域改革。深化医药卫生体制改革，继续推进公立医院综合改革，加快武夷总医院建设，鼓励组建医疗联合体，推广政和托管模式，推动形成分级诊疗制度。调整优化医疗机构布局，鼓励和引导社会资本办医。巩固完善基层医疗卫生机构运行新机制，加强疾病预防、妇幼健康等公共卫生服务。理顺学校管理体制，发挥名校带动效应，推动优质教育资源共享。深化文化事业单位体制改革，激发文化创新活力。深化财税体制改革，全面实施新预算法，推行预算绩效管理，推进财政国库集中支付改革。整合部门专项资金，提高财政性资金使用效益。推行“指挥部－总业主－代业主”工作机制，发挥市属国有企业在基础设施、城市、产业园区、旅游景区建设等方面作用。

　　提升对外开放合作水平。主动融入“一带一路”战略，推动“万里茶道”沿线城市文化传承、经贸合作、旅游协作。主动研究对接福建自贸区政策，促进投资贸易便利化。创新对台产业合作模式，广泛开展对台文化、教育、卫生、科技、体育交流合作。筹备召开第八届世界福建同乡恳亲大会，发挥南平旅港、澳门联谊会和闽北各地商会的作用，拓展与港澳地区经贸交流合作。积极参与周边地区经贸协作，推动建立更加紧密的合作机制。鼓励重点出口企业做大做强，扩大外贸出口。

　　（八）加强生态文明建设，扩大绿色发展后发优势

　　加强生态环境保护。积极创建国家级生态市，争取武夷山、邵武、光泽、松溪通过国家考核验收。完成植树造林31.3万亩。加快316国道绿色生态示范路建设，建成20条农村公路生态示范路。推进生态茶园建设，治理水土流失面积20万亩。加大阔叶林和珍贵树种的保护力度，探索生态林赎买机制。加快推进低丘缓坡地开发利用，严守耕地保护红线。开展矿山生态环境恢复和“矿山复绿行动”，巩固“青山挂白”治理成果。

　　持续推进节能减排。完善节能减排工作机制，构建排污权交易平台，加快18个节能减排示范项目建设，继续筛选一批示范项目，实施一批节能减排重点工程，确保年度节能减排目标顺利完成。严把新上项目产业政策、资源消耗和环境保护审批关，健全落后产能退出机制。加强水资源“三条红线”管理，积极推广节水技术和产品。扎实推进一批循环经济示范园区和示范项目，加快循环经济示范城市建设，争创国家级可持续发展实验区。

　　抓好环境综合整治。落实流域保护“河长制”，继续开展闽江流域水环境综合整治和农村环境综合整治，持续打击河道非法采砂，强化饮用水源保护、畜禽养殖面源污染整治、工业污染整治，加快工业园区和城乡污水垃圾处理设施建设。开展打击违法排污企业保障群众健康环保专项行动，重点开展大气污染、重金属污染、噪声污染以及环境风险隐患排查，提高环境监管能力。继续推进强制性清洁生产，完成一批企业的审核评估验收工作。

　　（九）提升公共服务水平，促进发展成果更多惠及民生

　　增强社会保障能力。实施企业用工调剂、大中专毕业生就业创业和服务基层计划，促进城镇困难人员和退役军人就业，新增城镇就业2.3万人，转移农村劳动力5.2万人。完善城乡居民基本养老保险和最低生活保障制度，完善企业欠薪应急保障金制度及企业工资保证金制度。持续推进城乡居民基本医疗保险政策一体化，启动实施城镇职工基本医疗保险基金市级统筹，完善以总额预付为主的定点医院医疗费用支付结算办法。加大棚户区、危旧房改造力度，建设保障性住房7924套。推动县级社会福利中心、乡镇敬老院建设全覆盖，建立健全农村留守儿童、妇女、老年人、失独家庭关爱服务体系，大力发展慈善和残疾人事业。

　　加快社会事业发展。全面推进素质教育，着力推进教育均衡化，实施薄弱校基本办学条件改善计划，完成义务教育标准化学校建设，新扩建10所公办幼儿园。扶持特殊教育，规范发展民办教育，重视继续教育和老年教育。支持武夷学院和高等职业院校发展，创新人才培养模式，深化校企合作，为绿色产业发展培育人才。加强师德师风教育，优化教师队伍结构。加强社会主义思想道德建设，打造具有时代特色的文化载体。整合基层公共文化资源，完善城乡公共文化服务体系。加强文化遗产的传承和保护，加快文化产业园区建设和文化产业龙头企业发展，打响朱子文化、茶文化、闽越文化等特色文化品牌，促进文化旅游融合发展。做好第二轮志书和年鉴编纂工作。全面贯彻人口和计划生育法，创新服务管理模式，提高出生人口素质。广泛开展全民健身运动，承办全国首届青运会排球赛。

　　加强社会治理创新。严格执行重大决策社会稳定风险评估机制，按照“路线图”依法处理信访事项，健全完善“大调解”工作体系。加强法制宣传和普法教育，做好“六五”普法检查验收。深化“平安南平”建设，创新立体化社会治安防控体系，加强网络安全管理，依法防范和打击违法犯罪活动。落实安全生产“党政同责、一岗双责”，持续开展安全生产标准化建设提升工程“三年行动”，加强重点行业重点领域专项整治，深化道路交通安全综合整治，开展火灾隐患排查治理。强化食品药品安全监管，持续治理“餐桌污染”，建设“食品放心工程”。落实党的民族政策和宗教工作基本方针。加强社区建设，做好村（居）委会换届选举工作。支持工会、共青团、妇联等群团组织开展工作。深化“双拥”共建，支持驻军部队建设，加强国防动员、国防后备力量和人民防空建设，力争蝉联全国“双拥模范城”。

　　三、加强政府自身建设

　　各级政府要深入贯彻依法治国基本方略，把政府工作全面纳入法治轨道，加快建设法治政府和服务型政府。

　　坚持依法行政，自觉维护法治权威。忠实履行宪法和法律赋予的职责，把党的领导贯彻到依法治市全过程。自觉接受人大、政协监督，重视社会和舆论监督，强化政府层级监督和监察、审计等专门监督。探索地方政府立法工作，全面推进覆盖城乡、优质均等的公共法律服务体系建设。建立行政机关内部重大决策合法性审查机制、重大决策终身责任追究制度及责任倒查机制，深化行政执法体制改革，严格规范公正文明执法。建立网上办事大厅，推进政务公开。加快政府职能转变，深化简政放权，建立权力清单、负面清单和责任清单，营造公平竞争环境。

　　坚持科学执政，提升服务发展能力。主动适应经济发展新常态，把握和尊重经济规律、社会规律、自然规律，认真学习研究政策，坚持理论培训和实践历练并举，拓宽知识面，更新知识结构，增强领导经济工作的专业化能力。围绕实施绿色发展战略，科学编制“十三五”规划，做好第三次全国农业普查前期准备工作。更加注重运用法治思维和法治方式来深化改革、推动发展、化解矛盾、维护稳定，提高领导经济工作的法治化水平。

　　坚持从严治政，切实改进工作作风。从严落实中央八项规定精神，持续整治“四风”，严格执行厉行节约反对浪费有关规定，基本完成机关公务用车制度改革。落实“三级联动、重心下移”工作机制，完善“民生110”网格化服务，形成更加直接、便捷、高效的联系服务群众机制。加强公务员队伍管理，深化机关效能建设，落实“马上就办”，治理“庸懒散奢”，强化行政问责，提高行政效能。全面落实党风廉政建设责任制，严格执行廉洁从政的各项规定，努力实现干部清正、政府清廉、政治清明。

　　各位代表，让我们更加紧密地团结在以习近平同志为总书记的党中央周围，在省委、省政府和市委的坚强领导下，紧紧依靠全市人民，进一步解放思想、开拓进取、奋勇拼搏，为全面完成“十二五”规划目标任务、加快推进科学发展跨越发展、建设“机制活、产业优、百姓富、生态美”的新南平而努力奋斗！
